# I'm Not Like the Others: Atypical Research Subjects in *JEAB* Publications

**DOI:** 10.1002/jeab.70047

**Published:** 2025-09-09

**Authors:** Eduardo J. Fernandez, Kennon A. Lattal

**Affiliations:** ^1^ School of Animal and Veterinary Sciences University of Adelaide Adelaide SA Australia; ^2^ Department of Psychology West Virginia University USA

**Keywords:** atypical subjects, behavior analysis, experimental analysis of behavior, laboratory research, species diversity

## Abstract

Comparative psychologists have been criticized for using a limited number of species in drawing general conclusions about broad behavioral processes. There are numerous examples, however, of the inclusion by behavior analysts of atypical subjects in their research. To examine the frequency and diversity in subject species used in the experimental analysis of behavior (EAB), *JEAB* publications between 1958 and 2023 were reviewed for their use of subjects other than pigeons, rats, humans, and nonhuman primates. Two hundred and twenty‐one occurrences of these atypical subjects were found across 204 articles, with 65 distinct species across both vertebrate and invertebrate taxa. The highest spikes in the frequency of atypical subject use occurred in the earliest and latest *JEAB* issues. The results are discussed in terms of the reasons for using diverse species, trends in use over time, and how EAB might benefit from continued, or even increased, diversification in the species used in its research.

## INTRODUCTION

A common criticism in the early days of comparative psychology by other animal behavior researchers, including Konrad Lorenz, was that few species were used for such behavioral comparisons (Burghardt, 2013). Some of the most well‐known texts in psychology and other disciplines concerned with the study of nonhuman animal behavior, including *Animal Intelligence* (Thorndike, 1911), *Conditioned Reflexes* (Pavlov, 1927), and *The Behavior of Organisms* (Skinner, 1938), derived their principles from research involving a single species (cat, dog, and rat, respectively). Likewise, Beach (1950) argued that comparative psychologists based almost all of their defining principles on a single subspecies: the albino rat (*Rattus norvegicus domestica*). As a result, questions about the generalizability of so‐called general process learning theories have occurred, based in part on limited species diversity of subjects (Domjan, 2000; Seligman, 1970). Therefore, some observers have argued that learning principles would benefit from an approach (and presumably experiments) involving a variety of taxa (Shettleworth, 1993; Timberlake, 1993).

Although the above may represent the norm both historically and currently, there are examples that deviate from such practices. For example, Bitterman (1960, 1965, 1976) used classical and operant conditioning procedures to compare behavioral processes across a variety of invertebrate and vertebrate species. Similarly, within the annals of the *Journal of the Experimental Analysis of Behavior* (*JEAB*), a diversity of species of subjects have appeared including octopuses (*Octopus vulgaris*; Dews, 1959), alligators (*Alligator mississippiensis*; Davidson, 1966), crows (*Corvus brachyrhynchos*; Powell & Kelly, 1976), elephants (*Elephas maximus*; Nissani et al., 2005), and crayfish (*Procambarus clarkia*; Gutierrez et al., 2022).

This tantalizing sample of species raises the question of just how diverse the species are that have appeared in the pages of *JEAB*. For example, Fernandez and Lattal (2025) found that the four most widely used species in research published in *JEAB*—pigeons, rats, humans, and other primates—account for about 94% of all species used in *JEAB*'s empirical research articles. The composition and distribution of the remaining 6% of species are the subject of this article.

## METHOD

Fernandez and Lattal (2025) described the methods used in this analysis. Atypical subjects (those classified by Fernandez and Lattal as belonging to their “Other” category) in *JEAB* publications between 1958 and 2023 were tallied. As in the earlier review, only empirical articles were considered. These were defined as reports of empirical findings derived from the experimental analysis of at least one live subject (virtual organisms and previously published data were excluded). In addition, numerical or graphical description of behavior was necessary for inclusion of the article in this review. Finally, any one article could count as more than one of the subject categories (instances) of an atypical subject, as when more than one species was included. For instance, Lubow (1964) used both goats and sheep as subjects. This article thus would be counted as one empirical article with two instances of an atypical subject.

### Interobserver agreement

Interobserver agreement (IOA) was calculated based on total agreement (Poling et al., 1995) for a combined category (nonhuman primates and other), as the comparison used the above combined category for their data set. A total of 20 of the 66 years examined, or 30% of all the data collected, were used to assess agreement. Agreement was determined by the first author functioning as the primary observer and comparing 19 of the 20 comparison years (2004–2022) based on data collected by the *JEAB* editorial staff (M. Galizio, personal communication, April 24, 2023). The twentieth and final (2023) comparison year was calculated by the second author. All IOA calculations ranged from 50% to 100%, with agreements below 80% occurring only when counts were below five articles (e.g., two and four articles counted for each observer, producing an agreement of 50%). Interobserver agreement for the Other category resulted in an average of 95.5%.

## RESULTS

**FIGURE 1:**
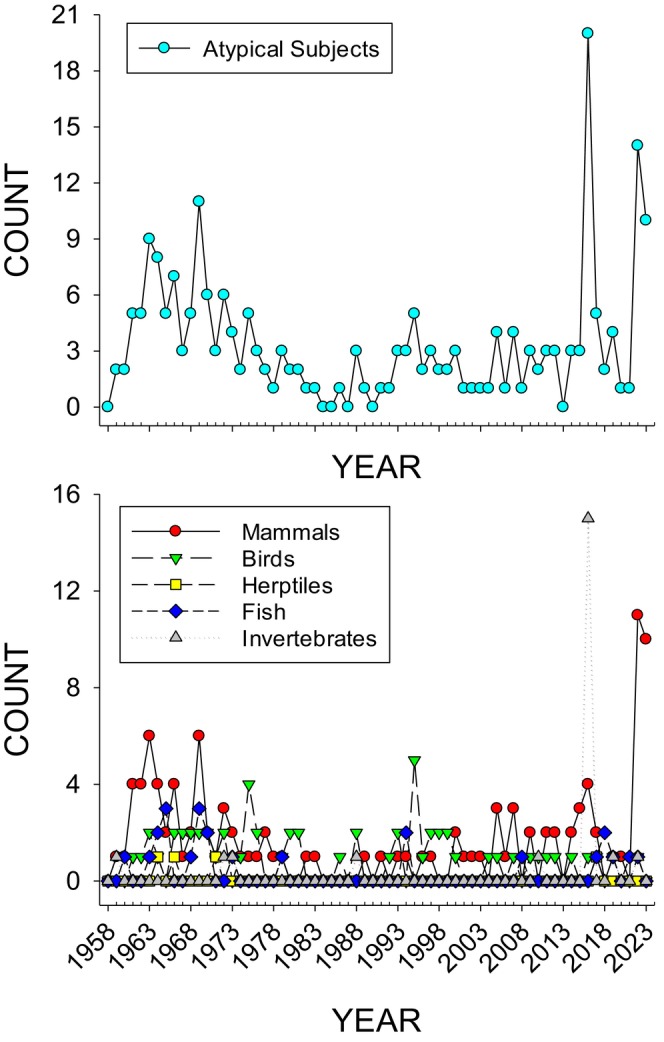
The frequency of total atypical subjects over time (year) are shown in the top graph, and the frequency per each taxonomic category appears in the bottom graph.

Figure 1 shows the year‐to‐year article count (frequency) of atypical subjects including their total and different taxa counts between 1958 and 2023. Different taxonomic categories used here and in subsequent figures are mammals, birds, herptiles, fish, and invertebrates.

At the beginning of the Journal (1958–1960), few atypical subjects were used (four articles total). From 1961 to 1975, with the exception of a few years (1967, 1971, 1973, and 1974), atypical subjects, primarily mammals (dogs or cats), appeared as subjects in five or more articles per year. From 1976 to 2015, except for 1995, 2005, and 2007, atypical subjects appeared as subjects three or fewer times per year. In the last decade, for three separate years (2016, 2022, and 2023), there were at least 10 occurrences of atypical subjects in publications per year, with 2016 showing five articles with 20 different occurrences of atypical subjects (primarily invertebrates [spiders]) and the latter 2 years having at least 10 articles with atypical subjects per year (primarily mammals [dogs]).

**FIGURE 2:**
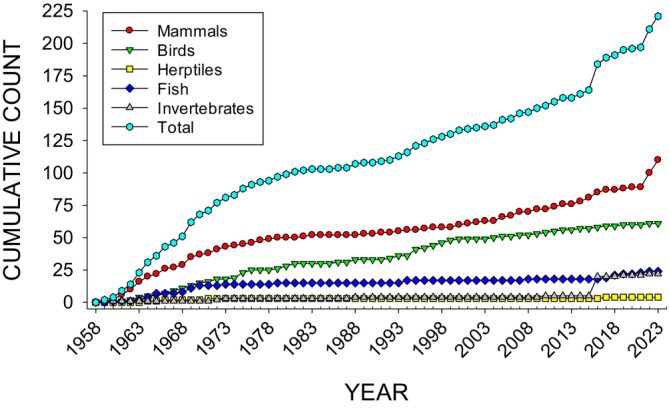
Cumulative count of atypical subjects in *JEAB* publications.

Figure 2 shows the cumulative article count of those reporting use of atypical subjects. This figure includes both total atypical subject numbers and different taxonomic categories to which they belong.

Two previously noted trends in atypical subject publications are emphasized by the cumulative graph: the early (1961 to 1975) and later (2016 to 2023) increase of the use of atypical subjects and the two spikes in atypical subject use (2016 and 2022–2023). The latter spikes for invertebrates in 2016 and mammals in 2022–2023 were primarily because of one article that included 15 species of spiders (Cross & Jackson, 2016) and the use of dogs as subjects in the later articles in a special issue on the experimental analysis of canine behavior and cognition (Hall et al., 2023).

**FIGURE 3:**
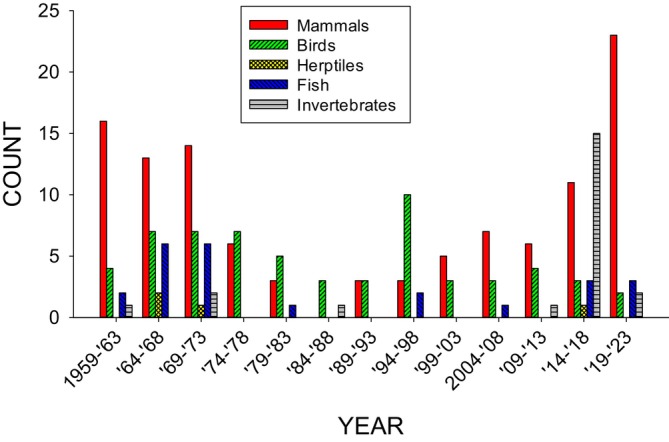
Five‐year count of atypical subjects in *JEAB* publications.

Figure 3 shows, in 5‐year blocks, the count of atypical subjects based on their taxonomic categories between 1959 to 2023 (1958 had 0 atypical subjects). This bar graph is in the same format for tracking subject use in *JEAB* publications as was used previously by Sokolowski et al. (2010) and Zimmermann et al. (2015).

In the early and later spikes of atypical subjects in *JEAB* publications, both primarily involved mammal species as subjects (dogs and cats in earlier years; dogs and mice in the later years). However, in four of the middle blocks (1974–1978, 1979–1983, 1984–1988, and 1994–1998), the most common atypical subjects were birds. Most of the 25 total occurrences of birds observed across these four blocks (20 years) were domestic chickens (*Gallus gallus domesticus*; 36%), domestic ducks (*Anas platyrhynchos domesticus*; 16%), or quail (either Japanese [*Coturnix japonica*] or bobwhite [*Colinus virginianus*]; 16%).

Table 1 shows the total number of articles and subjects where an atypical subject appeared, the breakdown of articles by subject taxa (mammal, bird, herptile, fish, or invertebrate), and the distinct number of species for both the total count and for each taxa.

**TABLE 1:** Atypical subjects (Other category) by taxa in *JEAB* publications (1958–2023).

Subjects	Count (Articles)	Count (Subjects)	Distinct species
*Total*	204	221	65
*Mammals*	107	110	21
*Birds*	61	61	15
*Herptiles*	4	4	3
*Fish*	24	24	5
*Invertebrates*	8	22	21

*Note*: As noted in the Methods, because more than one species or subject category type (instance) could occur in an article, this resulted in a greater total number of subjects relative to the number of articles.

In the 204 articles in which atypical subjects were used, there were 221 occurrences involving 65 distinct species. Atypical mammal subjects made up approximately half of those subjects (110 of 221) and almost a third of all the distinct species. Invertebrates made up almost another third of the distinct species, largely because of one article that used 15 distinct species of spiders (Cross & Jackson, 2016). The most frequently used atypical subject species were dogs = 30 articles, chickens = 25 articles, mice = 23 articles, cats = 13 articles, and goldfish = 10 articles.

## DISCUSSION

Atypical subjects were used in *JEAB* articles more frequently in the early and most recent issues, with 3 years of the last decade having some of the largest spikes in such use. The 2016 increase was largely due to one study using 15 different spider species; the last two years of spikes were because of special issues focused on atypical subjects, one on applied animal behavior (Alligood et al., 2022) and another on canine behavior and cognition (Hall et al., 2023).

One limitation of the present analysis was in the nonhuman primate category. Although primarily focused on primates used in laboratory settings, occasionally this category included nonlaboratory subjects not specifically included in the Other category. For example, Diamond et al. (2016) studied zoo orangutans. Another limitation was that the empirical article criteria required that an article include quantitative data, which was often not the case for technical reports. Waller (1960), as well as Rubadeau and Conrad (1963), for example, described operanda for use with dogs and insects, respectively, but included no data on such use, which excluded them from consideration in this review. Therefore, with respect to both limitations, there is some underreporting of the number of *JEAB* publications addressing issues related to atypical species use in EAB research. Even considering these omissions, however, the numbers reported would not change appreciably. Another limitation was that the third‐most represented atypical subject species, the laboratory or house mouse (*Mus musculus*), is a common laboratory animal. On one hand, it could be argued that they are less representative of a “true” atypical subject and their inclusion leads to overreporting the number of *JEAB* publications using atypical subjects. On the other hand, although they are typical subjects in other areas of psychology, in the context of EAB the present data suggest that their use is, in fact, atypical.

The criticism noted in the introduction that comparative psychology research focuses on a limited range of species can be applied to EAB, begging the question of why the focus of EAB research has been so narrowly on rats, pigeons, and primates. For most of these species, particularly the common pigeon (*Columba livia*) and rat (*Rattus norvegicus*), they are considered synanthropic species, meaning they live in close proximity to human populations (Klegarth, 2017). This has likely influenced their selection as research subjects. Rats historically and stereotypically are the experimental subject of choice by experimental psychologists, and so it probably was with Skinner in the beginning. The impetus for Skinner introducing pigeons into his science began with a fortuitous observation of “a flock of birds while travelling on a train” (Skinner, 1979, p. 241) that prompted him to ask “[c]ould they not guide a missile?” (p. 241). His musings led to Project Pigeon (Skinner, 1960), which required an organism with keen visual acuity and “imperturbability in the face of acceleration and noise” (Capshew, 1993, p. 843). Other reasons for selecting pigeons may have been their hardiness, longevity, and wide availability (Fernandez & Lattal, 2025). Skinner and others have described his discovery of shaping with pigeons and their subsequent use as laboratory subjects (Capshew, 1993; Fernandez & Martin, 2021; Peterson, 2004; Skinner, 1960). In addition and as Boakes (2023) noted, there may also have been a conceptual reason. Skinner's (1938) goal was to create an independent science of behavior, where the focus truly was on behavior and not physiology: “To use pigeons was a further departure from the tradition of experimental medicine in which Pavlov had been trained” (p. 226). Boakes also noted that rats (and presumably primates) were used because the mammalian brain structure of these species had similarities to that of other mammals, including humans, whereas the pigeon brain has an entirely different structure. Using pigeons as the primary subject suggested the placing of greater emphasis on environmental factors over neurological ones in the study of behavior. Skinner's research historically emphasized the importance of behavioral similarities across species rather than differences, suggesting another important reason for the intensive study of the behavior of a few representative species—the typical species mentioned throughout this review. Other reasons for focusing on rats and pigeons in particular are practical. As already noted, pigeons and rats are easily accessible and the details of their care and maintenance are well established. Pigeons in particular are hardy animals with a lifespan of 20 years in some cases. Apparatus for their investigation is standardized and widely available, saving investigators considerable startup time when undertaking new projects. In contrast, atypical species require specialized, sometimes costly, apparatus and often considerable time to establish a technology for their care, motivation, and maintenance. Much, but not all, of the research on atypical subjects in *JEAB* has been of a proof‐of‐concept nature, designed to demonstrate the feasibility and generality of operant conditioning principles using species other than the typical *JEAB* subjects.

### Atypical subjects and future EAB research and applications

Within the last several years, use of rats and pigeons as subjects in experiments reported in *JEAB—*and of pigeons in particular—has decreased concomitantly with an increase in the use of humans (Fernandez & Lattal, 2025). This may be due in part to the increased cost and regulations imposed on typical laboratory animals as well as to the decrease in funding for basic nonhuman animal research. This change perhaps has stimulated research with virtual organisms (for example, see McDowell, 2023), inexpensive‐to‐maintain species like fish (e.g., Elcoro et al., 2008) and invertebrates (Abramson & Wells, 2018), and research on topics relevant to applied animal behavior. The use of virtual organisms, although interesting from a behavioral modeling perspective, has limited utility in answering questions about the generalizability of learning principles across species. Additionally, many examples of the latter applied animal behavior research appear in the two previously mentioned *JEAB* special issues. These issues contain articles based on research conducted in such diverse nonlaboratory settings as homes, zoos, and animal shelters. There have also been calls for greater synthesis between EAB and applied animal behavior research and applications (e.g., Lattal & Fernandez, 2022), including that conducted in zoos (Fernandez & Timberlake, 2008; Lukas et al., 1998; Maple & Segura, 2015). The use of atypical subjects in both laboratory and nonlaboratory settings provides a unique opportunity to test the robustness and generality of behavioral principles. This suggests that this potential increasing trend in using a diversity of species and settings to study EAB will benefit both *JEAB* and, by extrapolation, a more general understanding of behavioral principles.

Arguments can also be made for the diversification of both species and settings in EAB. As noted above, fish and insects can be more cost effective to house and maintain, which has become an increasing concern for many animal laboratories (Baker & Kearney, 2015). There also are recent publications describing research designed to examine core behavioral phenomena that have used fish and invertebrates as subjects. Examples include recent research on delay of reinforcement and stimulus control (Kuroda et al., 2022; Scienza et al., 2019). Arguments have also been made for using insects, including various species of bees and wasps, to better understand the evolutionary processes involved in animal cognition (Simons & Tibbetts, 2019). Thus, both understudied taxa could provide promising and financially efficient models for future laboratory research into fundamental behavioral processes.

Diversification of subject settings including zoos, aquariums, farms, and shelters, could also use atypical subjects advantageously. Some of the first applications of EAB principles outside the laboratory were by students of Skinner, Keller and Marian Breland, and in the field of animal training and management that tradition continues today (Bailey & Gillaspy, 2005; Breland & Breland, 1951; Fernandez & Martin, 2021). As noted above, in the past several years, special issues focused on both applied animal behavior and domestic dogs have appeared among other more conventional topics in *JEAB*. There is increased emphasis on animal welfare, which ultimately includes the use of behavior as a key metric for assessing and improving the lives of animals in many environments, from homes to zoological parks (Dawkins, 2004; Hemsworth et al., 2015). Behavioral procedures are often used in evaluations of an animal's quality of life. Two examples are stimulus preference tests and progressive‐ratio schedules (Patterson‐Kane et al., 2008; Turner et al., 2024). In addition, Patterson‐Kane et al. (2008) reviewed the use of operant conditioning procedures as they have been applied to farm settings, where the application of basic laboratory procedures has contributed to evaluating production animal welfare. In one recent application, operant conditioning procedures were used to toilet train domestic cows to urinate on indoor pads, reducing the cows' environmental impact (Dirksen et al., 2021). Finally, EAB principles have been applied to the management of pests, including the control of invasive species in zoos that otherwise could damage ongoing breeding and ex situ conservation efforts of endangered species (Fernandez & Martin, 2023; Forthman & Ogden, 1992). In another arena, giant African rats have been trained using operant conditioning procedures on many tasks that benefit humans, including the detection of land mines and tuberculosis in humans (Edwards et al., 2015; Poling et al., 2015).

As a final note, traditional laboratory (EAB) concepts could benefit greatly from their implementation with atypical subjects. For example, contrafreeloading has been used to examine potential applications such as stimulus novelty and environmental enrichment with exotic animals such as giraffes (*Giraffa camelopardalis*; Sasson‐Yenor & Powell, 2019), maned wolves (*Chrysocyon brachyurus*; Vasconellos et al., 2012), and grizzly bears (*Ursus arctos horribilis*; McGowan et al., 2010). In addition, the testing of contrafreeloading across diverse species has led to questions about its generalizability, including preference for free food found in both domestic cats and dogs (Delgado et al., 2022; Rothkoff et al., 2024).

## CONCLUSION

Although the common pigeon remains a frequently research subject in *JEAB* (Fernandez & Lattal, 2025; Zimmermann, 2015), there was a time not long ago when this now‐typical subject was far less typical. It was Skinner's use of pigeons for applied wartime efforts that ultimately potentiated the selection of the pigeon as a “standard” research subject (Capshew, 1993; Skinner, 1960). Future innovative work, including the creation of apparatus and research methods, similarly could transform today's atypical subjects into regularly examined ones in EAB. Such a transformation will depend on how researchers select future research subjects and settings. Perhaps this review will encourage ensuing, and at least some present, researchers to think outside the box when deciding which species to put in it.

## AUTHOR CONTRIBUTIONS

The authors contributed equally to this project.

## CONFLICT OF INTEREST STATEMENT

None of the authors have a conflict of interest to disclose.

## ETHICS APPROVAL

No human or animal subjects were used to produce this article.

## Data Availability

The data that support the findings of this study are available from the corresponding author upon reasonable request.
